# Progressive multifocal leukoencephalopathy associated with thymoma with immunodeficiency: a case report and literature review

**DOI:** 10.1186/s12883-018-1041-4

**Published:** 2018-04-10

**Authors:** Tatsuya Ueno, Nobuyuki Sato, Tomoya Kon, Rie Haga, Jin-ichi Nunomura, Kazuo Nakamichi, Masayuki Saijo, Masahiko Tomiyama

**Affiliations:** 1 Department of Neurology, Aomori Prefectural Central Hospital, 2-1-1 Higashi-Tsukurimichi, Aomori, 030-8551 Japan; 2 Department of Thoracic Surgery, Aomori Prefectural Central Hospital, Aomori, Japan; 3 Department of Virology 1, National Institute of Infectious Diseases, Tokyo, Japan

**Keywords:** Progressive multifocal leukoencephalopathy, Good’s syndrome, Myasthenia gravis, Thymoma, Immunodeficiency, JC virus

## Abstract

**Background:**

The development of progressive multifocal leukoencephalopathy (PML) is associated with severe cellular immunosuppression. Good’s syndrome (GS) is a rare immunodeficiency syndrome related to thymoma, with the development of humoral as well as cellular immunosuppression; however, there are few reports of PML due to GS. One report suggested that the neurological symptoms of PML related to thymoma may be improved by a reduction of immunosuppressive therapy for myasthenia gravis (MG). It is therefore necessary to identify the cause of immunodeficiency in patients with PML to enable an appropriate treatment strategy to be adopted.

**Case presentation:**

A 47-year-old Japanese woman was admitted with aphasia and gait difficulty. She had an invasive thymoma that had been treated with repeated chemotherapy, including cyclophosphamide. She had also previously been diagnosed with MG (Myasthenia Gravis Foundation of America clinical classification IIa), but her ptosis and limb weakness had completely recovered. On admission, neurological examination revealed motor aphasia and central facial weakness on the right side. Laboratory studies showed severe lymphopenia, decreased CD4+ and CD8+ T cell and CD19+ B cell counts, and reduced levels of all subclasses of immunoglobulins, suggesting GS. Serology for human immunodeficiency virus (HIV) infection was negative. Brain magnetic resonance imaging showed asymmetric multifocal white matter lesions without contrast enhancement. Cerebrospinal fluid real-time polymerase chain reaction for JC virus was positive, showing 6,283,000 copies/mL. We made a diagnosis of non-HIV-related PML complicated with GS and probable chemotherapy-induced immunodeficiency. She then received intravenous immunoglobulin therapy, mirtazapine, and mefloquine, but died of sepsis 46 days after admission.

**Conclusions:**

It is necessary to consider the possibility of immunodeficiency due to GS in patients with PML related to thymoma. Neurologists should keep in mind the risk of PML in MG patients with thymoma, even if the MG symptoms are in remission, and should thus evaluate the immunological status of the patient accordingly.

## Background

Progressive multifocal leukoencephalopathy (PML) is a rare and potentially fatal demyelinating disease of the central nervous system caused by reactivation of the JC virus (JCV) [1]. The development of PML is associated with severe cellular immunosuppression e.g., human immunodeficiency virus (HIV) infection, and immunosuppressive therapy, including natalizumab and other monoclonal antibodies, fingolimod, and dimethyl fumarate [2].

Patients with thymoma often also have an autoimmune disease, such as myasthenia gravis (MG) [3]. Good’s syndrome (GS), also referred to as thymoma with immunodeficiency, is a rare immunodeficiency syndrome related to thymoma, characterized by hypogammaglobulinemia, low or absent B cells, CD4+ lymphopenia, and reversal of the CD4/CD8 ratio [4, 5]. GS thus involves not only humoral, also cellular immunosuppression [4, 5]. The incidence of hypogammaglobulinemia in patients with thymoma is 6%–11%, while GS occurs in 7% of patients with primary antibody deficiency referred to a chest clinic, and is the underlying cause of immunodeficiency in 1%–2% of patients with primary antibody deficiency receiving immunoglobulin replacement therapy [4, 6]. MG has been identified in 9.1%–15.7% of patients with GS [4, 5].

To the best of our knowledge, eight cases of PML with thymoma have been reported in the literature (Table 1) [7–14], though GS was confirmed in only three of these eight cases [11, 12, 14]. In the case of PML related to thymoma, it may be difficult to judge if the immunodeficiency is caused by chemotherapy for the thymoma, by GS caused by the thymoma itself, by immunosuppressive therapy for MG, or by a combination of these. The neurological symptoms improved in one patient with PML related to immunosuppressive therapy for MG, complicated with thymoma, following a reduction of immunotherapy for PML (Table 1) [10]. It is therefore important to identify the cause of the immunodeficiency responsible for PML, to enable the development of an appropriate treatment strategy. We herein report a patient with invasive thymoma who developed PML, who also had chemotherapy-induced immunodeficiency and GS due to an invasive thymoma.

**Table 1:** Clinical features of patients with progressive multifocal leukoencephalopathy related to thymoma

Reference	Age	Sex	Causes of immunodeficiency	Time	Treatment ofthymoma	JC virus in the CSF	CD19+ B cell (cells/μL)	CD4/CD8	CD4+ T cell (cells/μL)	CD8+ T cell (cells/μL)	Ig level (mg/dL)	PML Treatment	Prognosis	Survival period
[14]	65	F	Good’s syndrome	0	SR	>1million copies/mL	No detectable	0.66 (1.13–3.93)	NR	NR	IgG: 570 (670–1450) IgA: 22 (88–450) IgM: 22 (27–210)	Mirtazapine Mefloquine	Death	Probably 5–6 M
[13]	39	M	Chemotherapy for thymoma	10 Y	SR + CT	9,200 gEq/μL	1 (100–800)	0.2 (0.9–2.8)	19 (450–1500)	113 (250–1000)	NR	Cidofovir	Death	NR
[12]	79	F	Good’s syndrome	8 Y	SR	9,258 pairs/mL	Absent	1.5	648	425	IgG: 619 (700–1600) IgA: <25 (70–400) IgM: <18 (40–250)	Cidofovir	Death	5 M
[11]	58	M	Good’s syndrome	5 M	SR	810 copies/mL	NR	0.6 (0.8–2.4)	NR	NR	IgM: 31 (40–300)	Cidofovir Risperidone IVIg	Alive	At least 11 M
[10]	44	F	Immunosuppressive therapy for MG	2 M	SR	Positive	NR	NR	NR	NR	NR	Reduction of immunotherapy	Alive	At least 19 M
[9]	58	M	Chemotherapy Good’s syndrome?	9 Y	RT + CT	NR	NR	NR	NR	NR	NR	Cytrabine	Death	13 W
[8]	41	F	Immunosuppressive therapy for MG	29 Y	RT + SR	NR	NR	NR	NR	NR	NR	Reduction of immunotherapy	Death	6 W
[7]	39	F	Immunosuppressive therapy for MG Good’s syndrome?	3 Y	RT	NR	NR	NR	NR	NR	NR	None	Death	5 M
Present case	47	F	Good’s syndrome Chemotherapy	23 Y	RT + SR + CT	6,283,000 copies/mL	3	0.6	11	19	IgG: 282 (870–1700) IgA: 58 (110–410) IgM: 15 (35–220)	Mirtazapine Mefloquine IVIg	Death	3 M

Time = period from the diagnosis of thymoma to the onset of PML. Numbers in parentheses indicate the reference range. Patients in references [9, 10] survived for at least 11 and 19 months, respectively, but their subsequent prognosis was unknown. Abbreviations. *CSF*: cerebrospinal fluid; *CT*: chemotherapy; *IVIg*: intravenous immunoglobulin; *M*: months; *MG*: myasthenia gravis; *NR*: not reported*; PML*: progressive multifocal leukoencephalopathy; *RT*: radiation therapy; *SR*: surgical resection; *W*: weeks; *Y:* years

## Case presentation

**Fig. 1:**
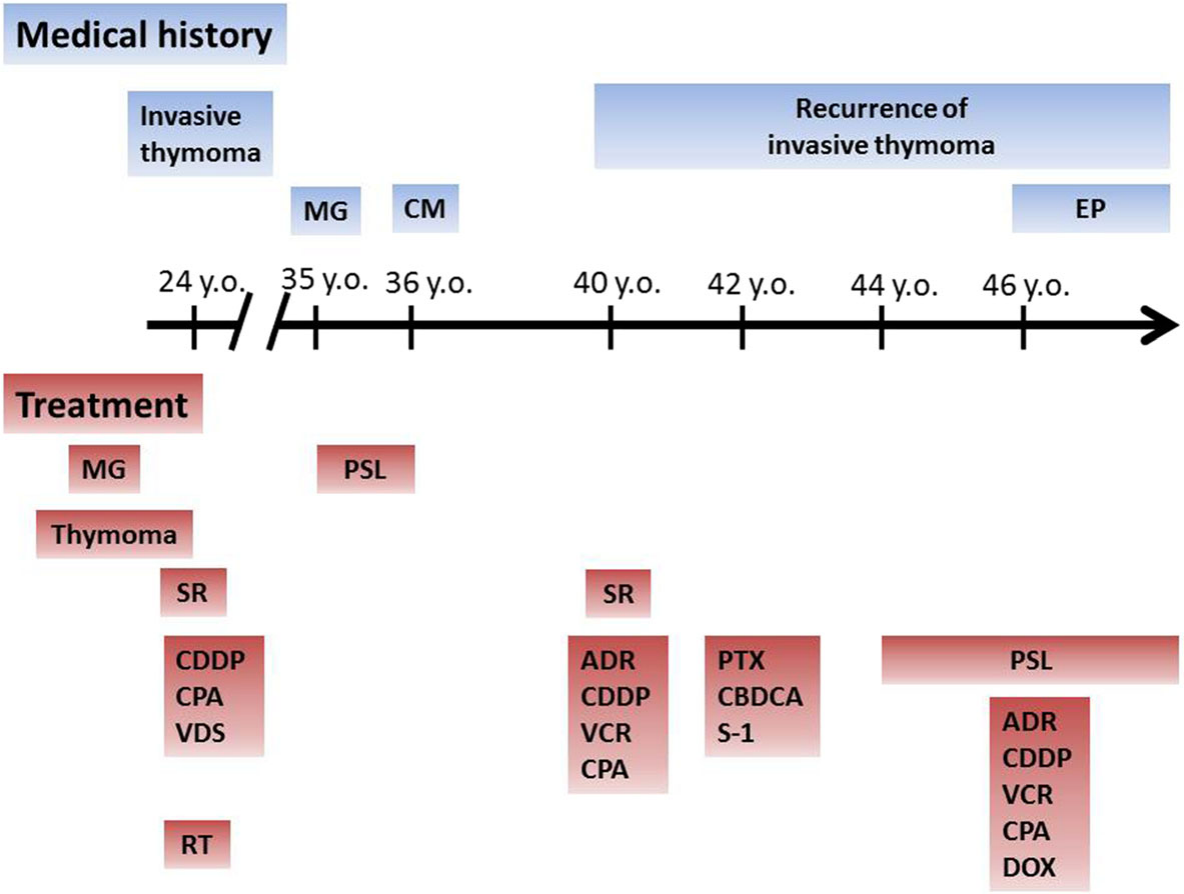
Medical history and treatment course. ADR: adriamycin; CBDCA: carboplatin; CDDP: cisplatin; CM: cryptococcal meningoencephalitis; CPA: cyclophosphamide; DOX: doxorubicin; EP: erythrodermic psoriasis; MG: myasthenia gravis; PTX: paclitaxel; PSL: prednisolone; RT: radiation therapy; S-1: tegafur/gimeracil/oteracil potassium; SR: surgical resection of thymoma; VCR: vincristine; VDS: vindesine

A 47-year-old Japanese woman with aphasia and gait difficulty was admitted for the evaluation of a brain magnetic resonance imaging (MRI) abnormality. She had gradually developed aphasia and gait difficulty 2 months before admission. Her medical history included an invasive thymoma diagnosed at 24 years old, MG (Myasthenia Gravis Foundation of America clinical classification IIa) at 35 years old, cryptococcal meningoencephalitis at 36 years old, and erythrodermic psoriasis at 46 years old. The time course of her medical history and treatments are shown in Fig. 1. Ptosis and limb weakness, as MG symptoms, had completely recovered. She demonstrated chemotherapy-induced immunosuppression, and the invasive thymoma (Masaoka stage IVB) was refractory to shrinkage by multiple chemotherapies. The invasive thymoma had also seeded intraperitoneally. She had received her last chemotherapy (cyclophosphamide, 500 mg; doxorubicin, 50 mg; cisplatin, 50 mg) 16 months before admission, and had been taking prednisolone (12.5 mg/day) from 44 years of age. Her family history was unremarkable.

On admission, her body weight was 39.5 kg and she was 151.7 cm tall (body mass index of 17.2 kg/m^2^). Her vital signs were normal and her Glasgow coma scale score was 12 (E4V2M6). Physical examination revealed widespread erythema with scaling and exfoliation of the skin due to erythrodermic psoriasis, and an invasive thymoma in the left lower chest wall. She had a central venous port for chemotherapy in her left upper chest wall. Neurological examination revealed motor aphasia and central facial weakness on the right side. Barré’s sign for the upper extremities and Mingazzini’s sign for the lower extremities were negative. Finger-to-nose and heel-to-knee tests demonstrated no abnormalities. Bilateral deep tendon reflexes were normal, but bilateral plantar responses were extensor.

**Fig. 2:**
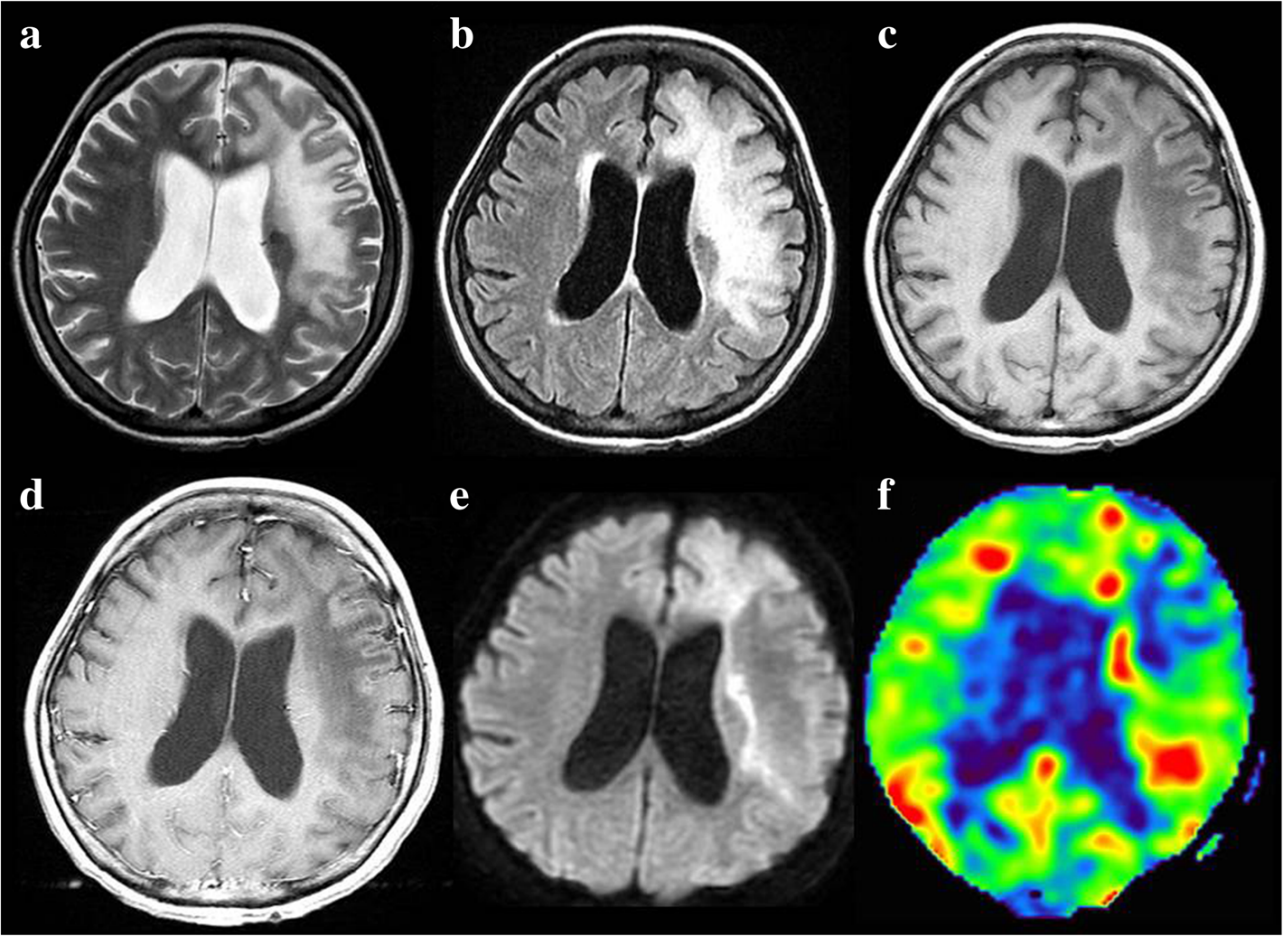
Brain magnetic resonance imaging on admission (**a**-**e**) and at 30 days after admission (**f**). T2-weighted images (**a**) and fluid-attenuated inversion recovery images (**b**) show hyperintense lesions in the left frontal white matter. These lesions were hypointense on T1-weighted images (**c**). There was no enhancement on gadolinium-enhanced T1-weighted images (**d**). Diffusion-weighted images (DWI) demonstrated a hyperintense lesion in the margin of the left frontal white matter (**e**). Arterial spin labeling revealed hyperperfusion in the DWI hyperintense lesion (**f**)

**Fig. 3:**
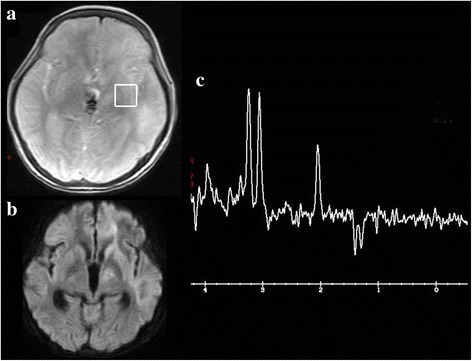
Magnetic resonance spectroscopy (MRS) and diffusion-weighted images (DWI) at 30 days after admission. Representative locations selected for MRS (**a**). MRS showed a decrease in N-acetylaspartate and increased lactate in the DWI hyperintense lesion (**b**, **c**)

**Fig. 4:**
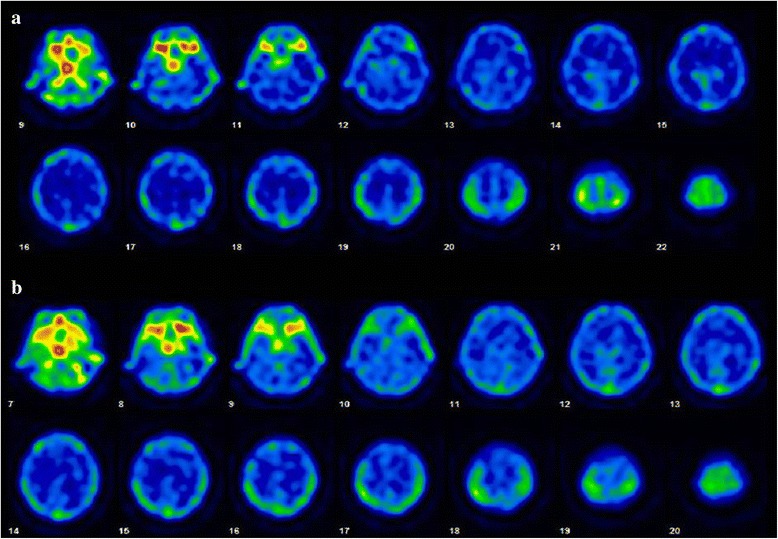
Thallium-201 single photon emission computed tomography at 11 days after admission. Early (**a**) and delayed (**b**) scans demonstrated no uptake in the lesions

Laboratory studies showed the following results: hematocrit, 32.6%; hemoglobin, 10.7 g/dL; red blood cell count, 3.74 × 10^6^/μL; mean corpuscular volume, 87.1 fL; platelet count, 22.7 × 10^4^/μL; and white blood cell count, 8.2 × 10^3^/μL, with 0.9% lymphocytes (74 cells/μL). Lymphocyte subgroups were as follows: CD4, 14.9% (normal range 25.0%–54.0%); CD8, 25.0% (normal range 2.0%–56.0%); CD4/CD8 ratio, 0.6; CD19, 0.4% (normal range 5.0%–24.0%); and CD20, 1.1% (normal range 3.0%–20.0%). Her total IgG level was 282 mg/dL (normal range 870–1700 mg/dL), IgA level was 58 mg/dL (normal range 110–410 mg/dL), and IgM level was 15 mg/dL (normal range 35–220 mg/dL). Serology for HIV type 1/2, human T-lymphotropic virus type 1, *Candida*, *Aspergillus*, *Toxoplasma*, and *Mycobacterium tuberculosis* were all negative. Anti-acetylcholine receptor (AchR) antibodies were positive (0.4 nmol/L: normal range < 0.3 nmol/L). Cerebrospinal fluid (CSF) analysis revealed a normal cell count, protein, and glucose, but an elevated IgG index (1.1) and elevated myelin basic protein levels (238 pg/mL). Oligoclonal bands and CSF cytology were negative. CSF cultures for bacteria, fungi, and *M. tuberculosis* were negative. CSF polymerase chain reaction (PCR) tests for *M. tuberculosis*, herpes simplex virus, varicella-zoster virus, Epstein-Barr virus, and cytomegalovirus were all negative, but CSF real-time PCR for JCV was positive, showing 6,283,000 copies/mL. Brain MRI showed hyperintense lesions in the left frontal white matter on T2-weighted images and fluid-attenuated inversion recovery images (Fig. 2a, b). These lesions were hypointense without contrast enhancement on T1-weighted images (Fig. 2c, d). Diffusion-weighted images (DWI) demonstrated a hyperintense lesion in the margin of left frontal white matter (Fig. 2e). Arterial spin labeling revealed hyperperfusion in the DWI hyperintense lesion (Fig. 2f). Magnetic resonance spectroscopy showed a decrease in N-acetylaspartate and increased lactate in the lesions (Fig. 3). Thallium-201 single photon emission computed tomography revealed no uptake in the lesions (Fig. 4). Based on the results of clinical features, imaging findings, and CSF PCR for JCV, the patient was finally diagnosed with non-HIV-related PML due to chemotherapy-induced immunodeficiency and GS [4, 15].

Monthly intravenous immunoglobulin (IVIg) therapy for hypogammaglobulinemia was started 10 days after admission. After the diagnosis of non-HIV-related PML, mirtazapine 15 mg/day was started 24 days after admission, and oral mefloquine 275 mg/day was also started for 3 days on day 25, followed by 275 mg once weekly. We also tapered her prednisolone to 10 mg/day on day 44. She had no deterioration of neurological symptoms indicating immune reconstitution inflammatory syndrome. However, she developed central venous port-related infections with no improvement of neurological symptoms, and received antibiotic and antifungal treatments on day 39. Although we tried to remove the central venous port, it could not be withdrawn without a risk of vascular injury. The above treatments were continued, but the patient unfortunately died of sepsis due to *Candida albicans* and *Staphylococcus epidermis*, as determined by blood culture on day 46.

## Discussion and conclusions

We present a patient with invasive thymoma-associated PML who also had chemotherapy-induced immunodeficiency and GS due to the invasive thymoma. This case demonstrates two important points: (1) it is necessary to consider the possibility of GS in patients with PML related to thymoma; and (2) neurologists should keep in mind the risk of PML in MG patients with thymoma, even if their MG symptoms are in remission.

According to our literature review, the possible causes of immunodeficiency in patients with thymoma-related PML include GS, chemotherapy-induced immunodeficiency, and immunosuppressive therapy for MG (Table 1). Our patient had CD4+, CD8+, and CD19+ lymphopenia, and hypogammaglobulinemia, which are characteristics of GS [4, 5]. These results suggest that this patient had GS. GS with PML has been reported in three previous cases, none of whom received chemotherapy for thymoma [11, 12, 14]; however, our patient had received repeated chemotherapy for invasive thymoma (Table 1). We were unable to judge if another two cases had GS, because their immunological statuses were not reported [7, 8]. Chemotherapeutic agents, such as cyclophosphamide, doxorubicin, vincristine, and methotrexate, can cause lymphopenia, including decreases in CD19+ B cells, CD4+ and CD8+ T cells, and decreased IgM levels with normal IgG levels [16]. The recovery periods for B cells and CD8+ T cells after chemotherapy are 1–3 months and 3–6 months, respectively [17–19]. The recovery of CD4+ T cells 6 months after completion of cytotoxic chemotherapy is inversely related to age for children younger than approximately 15 years, whereas persistent depletion is observed in older patients [20]. Given that a year had passed after the last chemotherapy in the current case, it was possible that her B and CD8+ T cells might have recovered, but that was not the case, while all immunoglobulin subclasses were also decreased. The patient’s age suggests that the decrease of CD4+ T cells may have been caused by her chemotherapy. There are currently no established diagnostic criteria or specific biomarkers for GS, and it is diagnosed on the basis of the existence of thymoma, hypogammaglobulinemia, low or absent B cells, CD4+ lymphopenia, and reversal of the CD4/CD8 ratio [4, 5]. Our patient with invasive thymoma had decreased B and CD8+ T cells, and hypogammaglobulinemia, even after considering chemotherapy, and we therefore considered the possibility of GS in addition to chemotherapy-induced immunodeficiency, resulting in severe humoral and cellular immunosuppression. To the best of our knowledge, this is the first reported case of PML with concurrent MG and GS against a background of thymoma. It is therefore necessary to judge patients with various possible causes of immunodeficiency, including chemotherapy, comprehensively, rather than assuming that chemotherapy-induced immunodeficiency is the cause.

There have been two reported cases of comorbid PML with MG (Table 1), neither of whom received chemotherapy. There was no description of any lymphopenia or hypogammaglobulinemia [8, 10] (Table 1), but their MG symptoms were presumably severe, because they received high-dose prednisolone and azathioprine for symptom control [8, 10]. It is therefore possible that this immunosuppressive therapy may have triggered the onset of PML. We were unable to judge if these two cases had GS. In the current case, PML occurred even when MG was in remission. However, this patient had severe humoral and cellular immunosuppression, and her MG symptoms may thus have improved. Neurologists should therefore consider the possibility of PML in patients with MG related to thymoma who experience progressive exacerbation of neurological symptoms, regardless of the severity of MG symptoms, and should evaluate the patient’s immunological status accordingly, including complete blood count, quantitative immunoglobulins, and B cell/T cell subsets. Regarding the need for immunological evaluations of MG patients without thymoma at the initial presentation, it is advisable to evaluate the immunological status of such patients if their neurological symptoms or anti-AchR antibody values deteriorate, given that a thymoma may be detected subsequently [21].

It is also necessary to consider treating GS to prevent the development of PML; however, this is challenging because there is currently no established treatment for GS [6], though thymectomy, radiotherapy, combination chemotherapy, immunosuppressive therapy, plasmapheresis, and splenectomy are all possible treatment options [4]. Squintani et al. reported on a patient with PML who had normal CD4+ and CD8+ T cells and hypogammaglobulinemia, indicating humoral but not cellular immunodeficiency [12]. Because PML is associated with severe cellular immunosuppression [2], hypogammaglobulinemia itself does not usually result in reactivation of JCV. Although Squintani et al.’s case might also have had cellular immunodeficiency, treatment of GS-related hypogammaglobulinemia by immunoglobulin replacement therapy may be considered for PML prevention.

The reported period from the diagnosis of thymoma to the onset of PML is 0–29 years (Table 1). Two patients with relatively short periods from thymoma diagnosis to PML onset survived [10, 11], including one patient with MG who received a reduction of immunotherapy for PML [10], and a patient with GS who received cidofovir and risperidone for PML and IVIg for GS [11]. No quantitative analyses were performed in the patient with MG [10], but the patient with GS had a low JCV level (810 copies/mL) [11]. In the case of thymoma diagnosis with simultaneous onset of PML, the patient with GS had a high JCV level (> 1 × 10^6^ copies/mL) and was treated with mirtazapine and mefloquine, but probably died within 5–6 months [14]. Patients with a low CSF JCV level were found to survive longer than patients with a higher JCV level [22], though the median survival of patients with non-HIV-related PML was only 3 months [23]. In the current case, the period from thymoma diagnosis to PML onset was 24 years. This patient had a high JCV level (6,283,000 copies/mL) and received mirtazapine, mefloquine, and IVIg, but died of sepsis after 3 months. JCV levels may thus be a better prognostic marker than time from thymoma diagnosis to PML onset in patients with PML related to thymoma; however, the rarity of reported cases means that this possibility needs to be evaluated in other cases.

In summary, we present a patient with invasive thymoma-associated PML who also had chemotherapy-induced immunodeficiency and GS due to thymoma. This case highlights the need to consider the possibility of immunodeficiency caused by to GS in patients with PML related to thymoma. Furthermore, physicians should keep in mind the risk of PML in MG patients with thymoma, even if their MG symptoms are in remission, and should evaluate the immunological status of the patients accordingly.

## Abbreviations

ADR: Adriamycin
CBDCA: Carboplatin
CDDP: Cisplatin
CM: Cryptococcal meningoencephalitis
CPA: Cyclophosphamide
CSF: Cerebrospinal fluid
CT: Chemotherapy
DOX: Doxorubicin
DWI: Diffusion-weighted images
EP: Erythrodermic psoriasis
GS: Good’s syndrome
HIV: Human immunodeficiency virus
JCV: JC virus
M: Months
MG: Myasthenia gravis
MRI: Magnetic resonance imaging
NR: Not reported
PCR: Polymerase chain reaction
PML: Progressive multifocal leukoencephalopathy
PSL: Prednisolone
PTX: Paclitaxel
RT: Radiation therapy
S-1: Tegafur/gimeracil/oteracil potassium
SR: Surgical resection of thymoma
VCR: Vincristine
VDS: Vindesine
W: Weeks
Y: Years
